# Supplementary material for: Perceived Diet Quality, Eating Behaviour, and Lifestyle Changes in a Mexican Population with Internet Access during Confinement for the COVID-19 Pandemic: ESCAN-COVID19Mx Survey
Source: Nutrients. 2021 Nov 26;13(12):4256. doi: 10.3390/nu13124256 (PMC8706190; doi:10.3390/nu13124256)
Supplement: Supplementary file 1 [file nutrients-13-04256-s001.zip › nutrients-1435413-supplementary.pdf]

Supplementary material 1. Survey on eating habits and lifestyle around the Coronavirus (Covid-19) pandemic in Mexico: ESCAN-COVID19Mx survey.

Perceived diet quality, eating behaviour and lifestyle changes in a Mexican population with internet access during confinement for COVID-19 pandemic: ESCAN-COVID19Mx Survey

Sophia E. Martínez-Vázquez* , Marena Ceballos-Rasgado, Rafael Posada, Claudia Hunot-Alexander*, Edna J. Nava-González, Ivonne Ramírez-Silva, Daisy Karina Aguilar-López, Gabriela Quiroz-Olguín, Beatriz López-Jara, Cristina Delgado-de-la-Cruz, Sol Huescas-Juárez, Mónica Silva, Martha Kaufer-Horwitz *

* Corresponding authors.

Encuesta sobre hábitos de alimentación y estilo de vida alrededor de la pandemia de Coronavirus (Covid-19) en México.

Selecciona la respuesta qué más se acerque a tus hábitos durante estos días que has permanecido en tu domicilio por la contingencia de Coronavirus (COVID-19). En algunas secciones se puede seleccionar más de una respuesta.

Sección 1. Datos generales

Contesta la siguiente información con tus datos generales

1. Sexo

- Femenino
- Masculino

Opción múltiple

2. ¿Qué edad tienes?

Texto de respuesta breve, número entre 18 y 99

3. ¿Cuánto pesas aproximadamente (kg)?

Texto de respuesta breve, número, es el número.

4. ¿Cuánto mides aproximadamente (p.e: 1.60)?

Texto de respuesta breve, número entre 0.90 y 2.10

5. ¿Cuál es tu escolaridad (nivel máximo de estudios)?

- Sin estudios
- Primaria/Secundaria
- Bachillerato
- Licenciatura
- Posgrado

Opción múltiple

6. ¿Actualmente cuántas personas residen en tu domicilio incluyéndote a ti?

Texto de respuesta breve, número entre 1 y 20

7. ¿Cuántas personas aportan ingresos económicos en tu domicilio?

Texto de respuesta breve, número entre 1 y 20

8. ¿Hay en tu domicilio menores de edad?

- Sí
- No

Opción múltiple

9. Indica tu código postal Si no lo conoces, consulta aquí http://www.correosdemexico.gob.mx/SSLServicios/ConsultaCP/Descarga.aspx

Texto de respuesta breve, número entre 00000 y 99999

Sección 2. Calidad de la alimentación

Contesta las siguientes preguntas que hablan sobre tu alimentación.

Todas son de opción múltiple

10. De acuerdo con el tiempo de aislamiento durante la contingencia y con tu ingreso, indica con cuál de las siguientes oraciones te identificas.

- No tengo ni tendré problemas económicos para comprar alimentos.
- Creo que puedo llegar a tener problemas económicos para comprar alimentos.
- Ya tengo problemas económicos para comprar alimentos.

11. ¿Consideras que tu alimentación era diferente ANTES de la contingencia?

- Sí (*va a la sección 2A, después continúa en sección 2 B*)
- No *(va a la sección 2B)*

Sección 2A. Contesta las siguientes preguntas que hablan sobre tu alimentación ANTES de la contingencia

Todas son de opción múltiple

12. ¿Bebías por lo menos 1.5 lts de agua natural al día?

- Sí
- No

13. ¿Consumías por lo menos, mínimo 200 g de verduras cocidas o crudas al día?

- Sí
- No

14. ¿Qué tipo de carne consumías con mayor frecuencia?

- Pollo
- Carne de res/cerdo
- Pescado
- No consumía carne

15. ¿Consumías pescado fresco o congelado (100 g) por lo menos una vez a la semana?

- Sí
- No

16. ¿Cuál era el tipo de grasa que consumías con mayor frecuencia en la semana?

- Aceites, aderezos y mayonesa
- Aceites, semillas y aguacate
- Mantecas, mantequillas, crema
- No sé

17. ¿Consumías por lo menos, 200 g de frutas al día?

- Sí
- No

18. ¿Consumías alimentos no preparados en casa 3 o más días a la semana?

- Sí
- No

19. ¿Consumías alimentos como frituras, embutidos, platillos empaquetados listos para calentar y servir) 2 o más días a la semana?

- Sí
- No

20. ¿Consumías postres o dulces comerciales 2 o más veces a la semana?

- Sí
- No

21. ¿Consumías leguminosas (haba, frijol, lenteja, garbanzo) por lo menos 3 días (300 g o 1 ½ tazas a la semana)?

- Sí
- No

22. ¿Qué tipo de cereales consumías con más frecuencia?

- Pastas, arroz, amaranto, avena, bolillo, telera o birote, tortillas
- Galletas, panes de dulce, pan de caja, tostadas fritas, pasteles empacados.
- No como trigo ni sus derivados

23. ¿Consumías 4 o más bebidas azucaradas (refrescos, aguas frescas con azúcar añadida) a la semana?

- Sí
- No

24. ¿Consumías al menos 200 ml de leche, yogurt, búlgaros o 100 gramos de quesos a la semana?

- Sí
- No

Sección 2B. Contesta las siguientes preguntas que hablan sobre tu alimentación DURANTE la contingencia

Todas son de opción múltiple

25. En la última semana ¿Has bebido por lo menos 1.5 lts de agua natural al día?

- Sí
- No

26. En la última semana ¿Has consumido por lo menos, mínimo 200 g de verduras cocidas o crudas al día?

- Sí
- No

27. En la última semana ¿Qué tipo de carne has consumido con mayor frecuencia?

- Pollo
- Carne de res/cerdo
- Pescado
- No consumo carne

28. En la última semana ¿Has consumido pescado fresco o congelado (100 g) por lo menos una vez?

- Sí
- No

29. ¿Cuál es el tipo de grasa que consumes con mayor frecuencia en la semana?

- Aceites, aderezos y mayonesa
- Aceites, semillas y aguacate
- Mantecas, mantequillas, crema
- No sé

30. En la última semana ¿Has consumido por lo menos, 200 g de frutas al día?

- Sí
- No

31. En la última semana ¿Has consumido alimentos no preparados en casa 3 o más días?

- Sí
- No

32. En la última semana ¿Has consumido alimentos como frituras, embutidos, platillos empaquetados listos para calentar y servir) 2 o más días?

- Sí
- No

33. En la última semana ¿Has consumido postres o dulces comerciales 2 o más veces?

- Sí
- No

34. En la última semana ¿Has consumido leguminosas (haba, frijol, lenteja, garbanzo) por lo menos 3 días (300 g o 1 ½ tazas a la semana)?

- Sí
- No

35. En la última semana ¿Qué tipo de cereales has consumido con más frecuencia?

- Pastas, arroz, amaranto, avena, bolillo, telera o birote, tortillas
- Galletas, panes de dulce, pan de caja, tostadas fritas, pasteles empacados.
- No como trigo ni sus derivados

36. En la última semana ¿Has consumido 4 o más bebidas azucaradas (refrescos, aguas frescas con azúcar añadida)?

- Sí
- No

37. En la última semana ¿Has consumido al menos 200 ml de leche, yogurt, búlgaros o 100 gramos de quesos?

- Sí
- No

38. De acuerdo a lo que acabas de responder, ¿consideras que tu alimentación era diferente antes de la contingencia?

- Sí, consumía más alimentos industrializados y comida rápida
- Sí, consumía más alimentos naturales y verduras
- No, era igual

Sección 2C. Responde lo siguiente

Todas son de opción múltiple

39. ¿Cuánto dinero destinas ahora a la compra de alimentos por semana?
    - Menos de 500 pesos
    - Entre 500 y 1,000 pesos
    - Entre 1,000 y 1,500 pesos
    - Entre 1,500 y 2,000 pesos
    - Más de 2,000 pesos

40. El gasto en tus alimentos a partir de esta contingencia ha sido
    - Menor al habitual
    - Igual al habitual
    - Mayor al habitual

41. El ingreso familiar derivado de la contingencia ha sido
    - Menor al habitual
    - Igual al habitual
    - Mayor al habitual

Sección 3. Estilo de vida

Responde las siguientes preguntas que se refieren a tu estilo de vida antes y durante la contingencia.

42. ¿Qué tan preocupada/o te encuentras sobre tu salud durante esta contingencia?
    - Muy preocupado
    - Preocupado
    - Poco preocupado

- Nada preocupado

Opción múltiple

43. ¿Cuáles son los medios de comunicación en los que te informas sobre temas de salud?

Puedes elegir varias opciones

- Redes sociales
- Páginas web
- Radio
- Televisión
- Periódicos
- Conversación con amigos/familiares/médicos

Casillas de verificación

44. ¿Cuáles son las fuentes de esos medios donde te informas sobre temas de salud?

Puedes elegir varias opciones

- Familiares, amigos
- Líder en salud / profesionales de la salud
- Programas de temas diversos
- Autoridades de gobierno y de instituciones de salud

Casillas de verificación

45. Selecciona la opción con la que más te identifiques:

- Durante este periodo de contingencia sigo haciendo mi vida normal
- Sólo salgo a la calle si tengo necesidad
- No salimos de casa, pedimos todo a domicilio

46. ¿Cuándo iniciaste el aislamiento voluntario en casa o cuarentena?

    (dd/mm/aa)

Fecha

47. Durante este tiempo de contingencia considero que:

- He disminuido de peso
- Mi peso se ha mantenido
- He aumentado de peso entre 1 y 3 kg
- He aumentado de peso más de 4 kg
- No estoy seguro que mi peso haya cambiado
- Actualmente estoy embarazada

Opción múltiple

48. Durante este tiempo de contingencia consideras que tu hábito de fumar:

- Empecé a fumar
- Ha disminuido
- Se ha mantenido igual
- Ha aumentado
- He dejado de fumar
- No fumo ni fumaba

Opción múltiple

49. Durante este tiempo de contingencia consideras que tu consumo de bebidas alcohólicas:

- Empecé a consumir bebidas alcohólicas
- Ha disminuído
- Se ha mantenido igual
- Ha aumentado
- He dejado de consumir bebidas alcohólicas
- No consumo ni consumía bebidas alcohólicas

Opción múltiple

50. ¿Cuántas horas duermes al día?

Texto de respuesta breve, número, entre 0 y 24

51. Durante la contingencia, ¿Se han modificado tus horarios para dormir?

Puedes elegir varias opciones

- No, siguen siendo los mismos
- Sí, me duermo más temprano
- Sí, me duermo más tarde
- Sí, mi sueño nocturno es interrumpido con más frecuencia
- Sí, duermo más siestas de lo habitual
- Sí, tengo un horario de sueño muy cambiante

Casillas de verificación

52. Durante la contingencia, ¿Se han modificado tus horarios para despertarte?

- No, siguen siendo los mismos
- Sí, me despierto más temprano
- Sí, me despierto más tarde
- Sí, me despierto varias veces en la noche

Opción múltiple

53. Actualmente ¿cuántas horas pasas frente a las pantallas al día desarrollando actividades laborales y no laborales (incluye celular, computadora, tabletas, TV)?

Texto de respuesta breve, número, entre 1 y 16

54. Consideras que el tiempo que pasas frente a las pantallas durante la contingencia:

- Es el mismo que antes
- Ha disminuido
- Ha aumentado ligeramente
- Ha aumentado considerablemente

Opción múltiple

55. ¿Consideras que durante la contingencia has permanecido más horas continuas sentado/recostado que antes?

- Sí

- No

Opción múltiple

56. ¿Consumes alimentos frente a las pantallas?

- No, con mi familia o solo(a) *(Pasa a Sección 4)*
- Sí, pero sólo 1 vez al día *(pasa a pregunta 57)*
- Sí, 2 a 4 veces al día *(pasa a pregunta 57)*
- Si, en todo momento *(pasa a pregunta 57)*

Opción múltiple

57. ¿Qué alimentos consumes frente a las pantallas? (escribe en la línea lo más frecuente ejemplo: papas fritas, fruta, sándwich, palomitas)

Texto de respuesta breve

Sección 4. Comer emocional

Responde las siguientes preguntas que se refieren a comer emocional

58. Por favor, lee las siguientes afirmaciones e indica la respuesta que más represente tu forma de comer en estos momentos

	**Muy en desacuerdo**	**En desacuerdo**	**Ni en acuerdo, ni en desacuerdo**	**De acuerdo**	**Muy de acuerdo**
Como MÁS cuando estoy ansiosa (o)					
Como MÁS cuando estoy enojada (o)					
Como MÁS cuando estoy preocupada (o)					

Como MÁS cuando estoy triste					
Como MÁS cuando estoy irritada (o) o enfadada (o)					
Como MENOS cuando estoy ansiosa (o)					
Como MENOS cuando estoy enojada (o)					
Como MENOS cuando estoy preocupada (o)					
Como MENOS cuando estoy triste					
Como MENOS cuando estoy irritada (o) o enfadada (o)					

Cuadrícula de opción múltiple, requerir una respuesta en cada fila.

59. De lo que acabas de responder, ¿crees que tus respuestas hubieran sido las mismas si hubieras contestado antes de la contingencia?

- No, hubieran sido diferentes
- Sí, hubieran sido las mismas

Opción múltiple

Sesión 5. Actividad Física

Contesta las siguientes preguntas en relación a tu actividad física antes y durante la contingencia.

60. En el mes previo a la contingencia ¿con qué frecuencia realizaste 30 minutos o más de actividad física que fuera suficientemente intensa? (Deporte o ejercicio estructurado).

- Varias veces al día
- Diario
- 5-6 veces por semana
- 3-4 veces por semana
- 1-2 veces por semana
- No realizaba actividad física intensa

Opción múltiple

61. Durante la contingencia, en la última semana ¿has realizado 30 minutos o más de actividad física que fuera lo suficientemente intensa? (deporte o ejercicio estructurado)

- Varias veces al día
- Diario
- 5-6 veces por semana
- 3-4 veces por semana
- 1-2 veces por semana
- No he realizado actividad física intensa

Opción múltiple

62. Antes de la contingencia ¿Cuánto tiempo pasabas sentado/acostado durante un día hábil?

Sin considerar tus horas de sueño nocturno

Texto de respuesta breve, número, entre 1 y 24

63. Durante la contingencia, ¿Cuánto tiempo pasas sentado durante un día hábil?

Texto de respuesta breve, número, entre 1 y 24

Has llegado al final de la encuesta, muchas gracias por tomar el tiempo para contestarla.

Si te interesaría ser contactado/a en el futuro en caso de que queramos hacer un seguimiento, podrías dejar tu correo a continuación

Texto de respuesta breve, no obligatoria.

En cursiva: formato de respuesta en Google Forms.
